# Investigation of the Microstructure and High-Temperature Performance of Laser-Clad Ni_50_(AlNbTiV)_50_-xY_2_O_3_ Complex-Concentrated Alloy Composite Coatings

**DOI:** 10.3390/ma18235303

**Published:** 2025-11-25

**Authors:** Wen Huang, Huaji Wang, Chunlei Li, Lei Li, Huan Yan, Wenyi Huang

**Affiliations:** 1 School of Materials, Shanghai Dianji University, Shanghai 201306, China; huangwen0620@163.com (W.H.); 17333636009@163.com (H.Y.); hwyi2024@163.com (W.H.); 2 China Helicopter Research and Development Institute, Jingdezhen 333001, China; 3 YTO Co., Ltd., Luoyang 471000, China; lichunlei202511@163.com; 4 School of Aeronautics, Shanghai Dianji University, Shanghai 201306, China

**Keywords:** laser cladding, complex concentrated alloy, microstructure, high-temperature performance

## Abstract

This study prepares Ni_50_(AlNbTiV)_50_-xY_2_O_3_ complex concentrated alloy composite coatings and analyzes their microstructure, phase composition, and high-temperature performance. The results indicate that each coating is composed of FCC phases, including γ-FeNi, Ni_3_Al, and Ni_3_(Al, Ti), as well as BCC phases such as Ni_3_Nb and Ni_3_Ti. With increasing Y_2_O_3_ content, the lamellar structure in the inter-dendritic regions of the coatings first decreases and then increases. The wear mechanism of the coating without Y_2_O_3_ addition under high-temperature conditions involves the combined effects of abrasive wear and adhesive wear. The coating with a 0.6 wt.% addition exhibits the smallest cracks and the lowest oxidation rate in high-temperature oxidation and thermal shock tests, with the oxidation weight gain reduced by 26.2% compared to the coating without Y_2_O_3_ addition. The abrasive and adhesive wear of this coating is mitigated, and the average friction coefficient and mass loss are decreased. When the mass fraction of Y_2_O_3_ is 0.4 wt.%, the coating structure shows a significant refinement effect and exhibits the best performance in tensile strength and high-temperature friction and wear tests.

## 1. Introduction

Laser cladding is an advanced manufacturing technique that utilizes a high-energy laser beam to melt and deposit metallic powder or wire onto a substrate surface, thereby forming high-performance coatings or repairing damaged components. Compared with conventional manufacturing technologies, laser cladding offers remarkable advantages in terms of material utilization efficiency, microstructural control, process flexibility, thermal influence minimization, and repair capability [1,2,3]. According to the morphology of the feedstock materials, laser-cladding alloys can be classified into three categories: powder, wire, and sheet. Among them, powder feedstock materials are the most widely used due to their mature preparation technology and broad applicability [4,5,6]. Within powder-based laser cladding materials, self-fluxing alloy powders, metal–ceramic composite powders, and complex concentrated alloy (CCA) powders have attracted significant attention because of their superior functional and mechanical properties. Self-fluxing alloy powders are generally categorized into Co-based, Fe-based, and Ni-based types, depending on the dominant constituent element. These powders typically consist of metallic matrices alloyed with various elements, possessing low melting points and excellent self-fluxing properties, which ensure good wettability and strong metallurgical bonding during the cladding process [7]. However, each material type also exhibits certain limitations. The thermal stability of self-fluxing alloys may degrade under high-temperature service conditions, leading to performance deterioration. In addition, the evaporation of low-melting-point elements may adversely affect the compositional homogeneity and mechanical integrity of the coating [8,9,10]. Metal–ceramic composite powders exhibit outstanding wear resistance and high-temperature performance, yet the significant mismatch in thermal expansion coefficients between the metallic and ceramic phases can induce severe thermal stresses, thereby increasing the risk of crack formation in the coating. Moreover, the incorporation of ceramic particles tends to reduce the overall plasticity and deformability of the coating, limiting its use in complex service environments [11,12,13]. In contrast, complex concentrated alloys (CCAs), as a new class of multi-principal-element metallic materials, have emerged as a prominent research focus in materials science since their introduction in the early 21st century. Unlike conventional alloys that rely on a single principal element, CCAs are composed of multiple major elements with near-equimolar or moderately varying concentrations—a design philosophy derived from the concept of high-entropy alloys (HEAs). CCAs exhibit remarkable advantages in structural applications at elevated temperatures, including high melting points, superior strength, ductility, and toughness [14,15].

Li et al. [16] prepared TiNiSiCrCoAl high-entropy alloy coatings on the surface of Ti-6Al-4V titanium alloy using laser cladding technology. The study found that the coatings mainly consisted of amorphous structures and σ phases. Moreover, with increasing laser scanning speed, the volume fraction of the amorphous structure in the coatings increased. After oxidation at 800 °C for 48 h, the coatings exhibited superior oxidation resistance compared to the Ti-6Al-4V alloy. Zhang et al. [17] prepared AlCrFeMnNi high-entropy alloy coatings on 316 L stainless steel surfaces. The study revealed that the laser-clad high-entropy alloy coatings had dense and uniform structures composed of a single BCC solid solution phase. At room temperature, the primary wear mechanism of the coatings was abrasive wear. As the temperature gradually increased, a dense oxide film formed on the coating surface, and the main wear mechanisms became oxidative wear and adhesive wear. At 400 °C, the coatings exhibited the best wear performance. Zhang et al. [18] investigated a novel refractory high-entropy alloy coating TiZrNbWMo, where the laser-clad coatings mainly consisted of body-centered cubic solid solutions and a small amount of precipitated phases. After annealing at 800 °C, 1000 °C, and 1200 °C for 20 h, the structure of the coatings remained essentially unchanged, indicating good thermal stability. Additionally, the coatings exhibited high microhardness, with the as-clad coatings reaching approximately 700 HV. After annealing, the hardness significantly increased, reaching up to 1300 HV, demonstrating excellent anti-softening performance. These studies illustrate that preparing high-entropy alloy coatings via laser cladding technology is an effective method to enhance the thermal stability of materials. Further reinforcement and refinement of CCA systems are imperative to ensure their reliable performance under increasingly harsh service environments.

In our previous research [19], ultrasonic surface-rolling strengthening technology was employed to apply periodic impact forces on the surface of Ni_50_(AlNbTiV)_50_ laser-clad coatings, refining surface grains and thereby improving the wear resistance and corrosion resistance of the coatings. However, this equipment is expensive, and processing complex precision-structural components is challenging. In recent years, numerous studies have focused on improving the coarse microstructure in high-entropy alloy systems by adding specific proportions of rare-earth elements, purifying the molten pool, and reducing the dilution rate between the cladding layer and the substrate to achieve higher component performance. For example, Zhang et al. [20] prepared TiC on Ti6Al4V titanium alloy surfaces and investigated the effects of Y_2_O_3_ addition on coating formation quality and microstructure. They found that adding 2 wt.% Y_2_O_3_ could completely eliminate cracks and significantly reduce porosity, thereby improving coating performance. Yang et al. [21] mixed H13 steel powder, WC powder, and Y_2_O_3_ powder to prepare coatings on 8407 steel surfaces, finding that the high-temperature performance of the coatings was mainly attributed to the presence of M_7_C_3_ composite strengthening phases, while the addition of Y_2_O_3_ accelerated the decomposition of WC particles, increased the types and quantities of carbides in the coatings, and refined the grains. Shi et al. [22] added different amounts of nano-La_2_O_3_ to Ni60A/SiC composite powders and found that hard secondary phases such as Cr_7_C_3_ and CrC formed in the composite coatings, promoting the enhancement of overall coating performance.

Based on the aforementioned research background, this study employs laser cladding technology to prepare Ni_50_(AlNbTiV)_50_-xY_2_O_3_ composite coatings with Y_2_O_3_ mass fractions of 0 wt.%, 0.2 wt.%, 0.4 wt.%, and 0.6 wt.%. The effects of different Y_2_O_3_ addition amounts on the coating microstructure are investigated, and the influences of varying Y_2_O_3_ additions on the coating microstructure and high-temperature performance are evaluated through high-temperature oxidation tests, thermal shock tests, and high-temperature friction and wear tests.

## 2. Experimental Materials and Methods

### 2.1. Experimental Equipment and Sample Preparation

**Figure 1:**
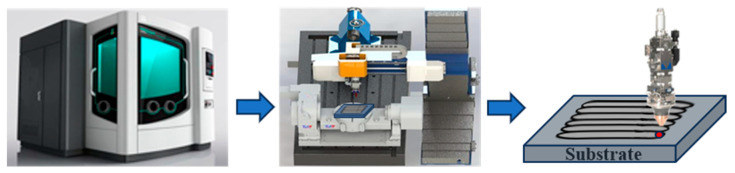
Schematic diagram of laser cladding equipment.

An LDM diode laser system from Germany’s Laserline was employed (Mülheim-Kärlich, Germany), with a laser power of 3000 W, a scanning speed of 4 mm/s, and an overlap rate of 50%. The schematic diagram of the equipment is shown in Figure 1. The cladding materials used were Ni_50_(AlNbTiV)_50_ high-entropy alloy powder prepared by vacuum atomization and high-purity Y_2_O_3_ rare earth oxide powder. The average particle size of the Y_2_O_3_ rare earth oxide was 10 μm. The Ni_50_(AlNbTiV)_50_ alloy powder had a particle size range of 53–150 μm and a chemical composition of 6.05% Al, 20.34% Nb, 10.90% Ti, 11.16% V, and the balance Ni (wt.%). The mixed powder was placed in a ball mill together with stainless steel balls of 5 mm diameter and subjected to ball milling at a rotation speed of 30 r/min for 20 min. Four Ni_50_(AlNbTiV)_50_-xY_2_O_3_ (x = 0 wt.%, 0.2 wt.%, 0.4 wt.%, and 0.6 wt.%) composite coatings were prepared, denoted as Y0, Y1, Y2, and Y3, respectively.

### 2.2. Microstructure and Phase Composition Test

The cross-section of the cladding layer was immersed in an etching solution (75% HCl and 25% HNO_3_) for 15 s. After etching, the microstructure of the samples was observed using a ZEISS MERLIN Compact SEM electron microscope (Oberkochen, Germany), and elemental analysis of the coatings was performed with an EDAX Apollo XP energy-dispersive spectrometer (EDS) probe (Mahwah, NJ, USA). The phase composition was determined using a Bruker D8 Advance X-ray diffractometer (Billerica, MA, USA).

### 2.3. High-Temperature Oxidation Test

The samples were placed in a non-vacuum tube furnace at 800 °C for heat preservation and subjected to an oxidation cycle experiment for 32 h. They weighed every 4 h using a high-precision electronic balance (with an accuracy of 0.0001 g).

### 2.4. Thermal Shock Test

The specimen was placed in a non-vacuum tube furnace at 800 °C for heat preservation treatment. After 10 min, it was taken out and cooled in water for 30 s. After drying, it was put back into the furnace for the next thermal fatigue test. This process was repeated for a total of 60 cycles.

### 2.5. Mechanical Properties Test

The microhardness of the coating was tested using the HXD-1000TMC/LCD micro-Vickers hardness tester (Wuxi Metes Precision Technology Co., Ltd., Wuxi, China), with a pressure load of 10 N and a dwell time of 10 s. The measurement points were set at intervals of 200 μm from the end of the coating surface towards the substrate, with 25 points in each group. Three groups of data were measured for each sample, and the average value was taken. The tensile stress–strain curve was measured by the CMT5105 tensile testing machine (SANS, Ningbo, China), at a tensile strain rate of 1 mm/min. High-temperature friction and wear tests were conducted using an MMQ-02G high-temperature friction and wear testing machine (Changsha Suny Electronic Technology Ltd., Changsha, China), with a Si_3_N_4_ pin of 6 mm diameter. The specific experimental parameters were as follows: load of 20 N, rotation speed of 200 r/min, friction radius of 18 mm, test duration of 10 min, and test temperature of 800 °C. SEM and a Zeiss Smart Proof 5 white light confocal microscope were used to measure the wear morphology of the samples.

## 3. Results and Discussion

### 3.1. Microstructure and Phase Composition

**Figure 2:**
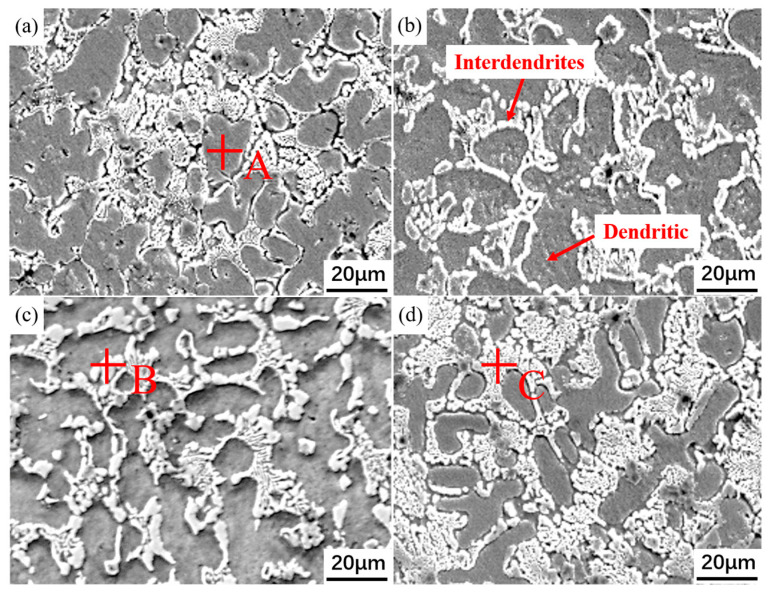
Microstructure morphologies of coatings with different Y_2_O_3_ additions: (**a**) Y0; (**b**) Y1; (**c**) Y2; (**d**) Y3.

Figure 2 shows the microstructure morphologies of the four composite coatings. It can be observed that the microstructures of each coating mainly consist of dendrites, white granular precipitated phases, and lamellar inter-dendritic structures. Comparison reveals that the Y0 coating exhibits a relatively coarser microstructure compared to the other coatings. With the addition of Y_2_O_3_, the coating microstructure is significantly refined, with the coating containing 0.4 wt.% Y_2_O_3_ showing the most pronounced refinement effect. However, as the Y_2_O_3_ addition increases to 0.6 wt.%, the coating microstructure gradually coarsens. These differences in microstructure arise because, during the solidification process of the molten pool, Y_2_O_3_ can act as heterogeneous nucleation cores, promoting the nucleation process. Heterogeneous nucleation lowers the nucleation energy barrier and increases the nucleation rate, thereby refining the coating microstructure [23]. When the Y_2_O_3_ addition reaches 0.6 wt.%, a sufficient quantity of dispersed Y_2_O_3_ particles can delay heat dissipation from the current position to the surrounding material during solidification, thereby reducing the cooling and solidification rates of the melt and leading to the formation of coarser grains [24,25].

**Figure 3:**
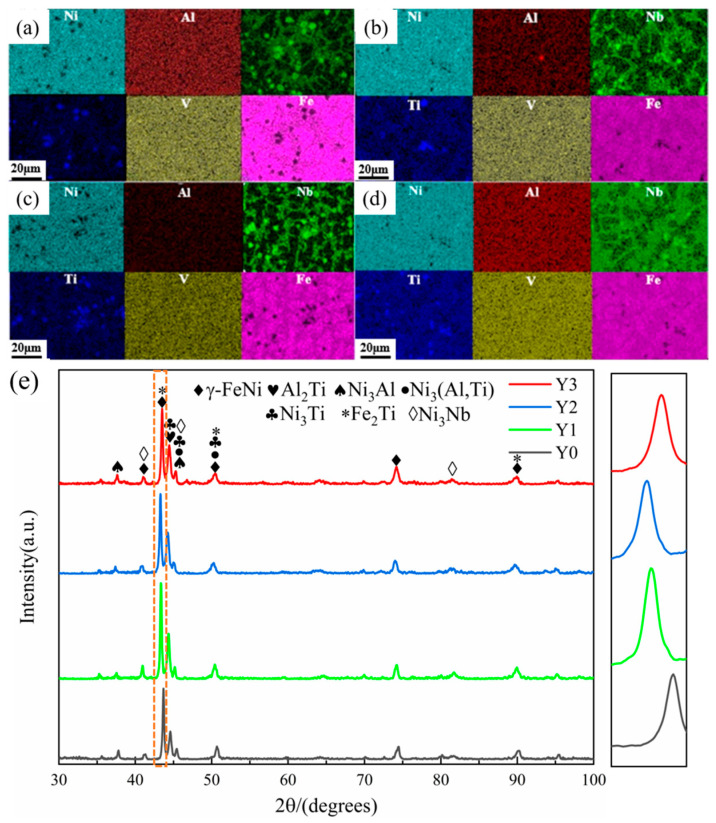
EDS area scan results and XRD scan patterns of the composite coatings: (**a**) Y0; (**b**) Y1; (**c**) Y2; (**d**) Y3; (**e**) XRD diffraction patterns of coatings with different Y_2_O_3_ additions.

Figure 3a–d shows the EDS scanning results of the four coatings. Combined with Table 1 and the XRD scanning results in Figure 3e, it is found that the dendritic core regions of each coating are rich in Fe and Ni elements, mainly in the form of FCC-structured γ-FeNi, Ni_3_Al, and Ni_3_(Al, Ti). This is because Fe and Ni elements have similar atomic radii and crystal structures, tending to co-precipitate and solidify preferentially, forming a relatively stable solid solution. In the inter-dendritic regions, Nb and Ti elements are significantly enriched, forming lamellar inter-dendritic compounds. During the solidification process, Nb and Ti elements have high melting points and large atomic radii, which reduce their solubility with other elements, making them more likely to be rejected and accumulate at the forefront of the solid–liquid interface. Additionally, the large atomic radii of Nb and Ti elements cause significant lattice distortions in the solid solution, promoting changes in the crystal structure. As a result, in a system with limited solid solubility, only a small amount of Nb and Ti elements can dissolve into the dendritic regions. On the other hand, the sluggish diffusion effect of high-entropy alloys further inhibits the diffusion of Nb and Ti elements, making them more likely to be rejected to the interdendritic regions and form enrichments [26]. The inter-dendritic regions of the Y0 and Y3 coatings are enriched with more lamellar compounds of Nb elements, while the dendritic boundaries of the Y1 and Y2 coatings are clearer, with fewer lamellar precipitates. This is related to the addition of excessive Y_2_O_3_. An appropriate amount of Y_2_O_3_ can purify the melt pool, refine the grains to a certain extent, and reduce interdendritic precipitates. However, the addition of excessive Y_2_O_3_ increases the solidification time and promotes the nucleation and growth of lamellar structures instead.

It is worth noting that the addition of Y_2_O_3_ did not change the phase composition of the coating but enhanced the peak intensity of the γ-FeNi phase. Moreover, due to the atomic radius of Y_2_O_3_ being larger than that of the metal atoms in Ni_50_(AlNbTiV)_50_, the lattice parameters increase after the addition of Y_2_O_3_, resulting in a decrease in the diffraction angle of the γ-FeNi phase and a leftward shift in the diffraction peak. This indicates significant changes in the internal grains of the material, with lattice distortion and grain refinement occurring in the composite coating.

### 3.2. High-Temperature Oxidation Behavior

**Figure 4:**
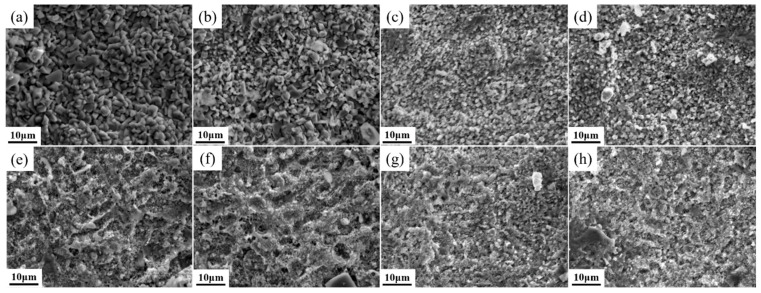
Surface oxidation morphologies at different positions of coatings with different Y_2_O_3_ additions: (**a**,**e**) Y0; (**b**,**f**) Y1; (**c**,**g**) Y2; (**d**,**h**) Y3.

Figure 4 shows the surface morphologies at different positions of the coatings after oxidation at 800 °C for 32 h. Comparison reveals that the oxidation products and pores in the coatings with Y_2_O_3_ addition are finer. With increasing Y_2_O_3_ content, the large-sized blocky clustered oxides on the coating surface gradually become smaller. Grain refinement plays a dominant role. Finer grains imply a greater grain boundary area, and grain boundaries are preferred sites for oxide nucleation. More nucleation sites enable the oxides to form in finer particle sizes, which helps improve the adhesion between the oxide layer and the coating matrix, maintaining the stability of the oxide layer [27,28]. In long-term high-temperature environments, larger oxide particles tend to grow continuously and form high stress concentrations. When the thermal stress exceeds the bonding strength between the coating material and the oxide film, it leads to loosening or cracking of the oxide layer [29,30]. These defects provide diffusion channels for oxygen ions, allowing them to easily penetrate through cracks and pores to the subsurface of the oxide film, further invading the exposed coating material.

**Figure 5:**
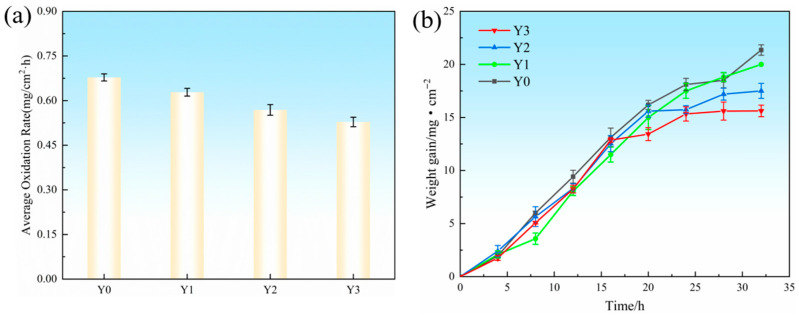
High-temperature oxidation test data of composite coatings at 800 °C: (**a**) oxidation rate; (**b**) oxidation weight gain curves.

Figure 5 presents the oxidation rate and oxidation weight gain curves of the composite coatings. As shown in Figure 5a, when the Y_2_O_3_ content increases to 0.6 wt.%, the coating exhibits the lowest oxidation rate. Figure 5b shows that all four coatings follow a similar oxidation weight gain trend during high-temperature oxidation. In the early oxidation stage, the weight gain of each sample increases linearly with time. With further prolongation of oxidation time, the oxidation weight gain curves gradually transition into a parabolic shape. This indicates that all samples exhibit a pronounced accelerated oxidation behavior at the initial stage, during which the oxide film continuously forms, cracks, and peels off. The exposed metal surface then reacts further with oxygen, leading to continuous increases in oxidation weight gain. As oxidation proceeds, the rate of weight gain gradually decreases, reflecting the reduction in oxidation kinetics, and the overall behavior follows a typical parabolic oxidation law. Among all coatings, the Y3 coating exhibits the smallest oxidation weight gain, approximately 16 mg/cm^2^, which is significantly lower than that of the other coatings, demonstrating its superior oxidation resistance. Combined with the microstructural analysis in Figure 2, it can be seen that the Y3 coating possesses a denser lamellar microstructure. At elevated temperatures, the oxidation products formed in the interdendritic regions enriched with Nb and Ti exhibit lower oxygen diffusion coefficients and smaller Gibbs free energies. For instance, the oxygen diffusion coefficient of TiO_2_ at 1000 °C is approximately 10^−12^ cm^2^/s, and the Gibbs free energy of Nb_2_O_5_ formation is about −1766 kJ/mol [31,32]. These characteristics contribute to the long-term thermal stability and excellent oxidation resistance of the Y3 coating under high-temperature conditions.

The formation of the oxide film primarily relies on chemical reactions. As the oxidation process continues, the growth of the oxide film on the coating surface gradually shifts to being dominated by the diffusion of elements from the interior of the coating material to the surface and the migration of ions within the oxide film. Compared to traditional alloys, Ni_50_(AlNbTiV)_50_ exhibits unique oxidation behavior due to its richer elemental composition and higher elemental content diversity. Additionally, the dilution effect during the laser cladding process leads to significant changes in the distribution and content of elements in the coating, thereby influencing the oxidation process. Therefore, the oxidation reaction of complex concentrated alloy composite coatings is not entirely controlled by the principal elements in the alloy. In the initial stage of the oxidation reaction, selective oxidation phenomena still exist. According to oxidation kinetics, the more stable the oxide, the more readily the corresponding element is oxidized. Elements that form oxides with lower formation enthalpies exhibit stronger spontaneity in reacting with oxygen, leading them to oxidize preferentially and form the oxide film [33,34]. However, the actual oxidation process is also controlled by ionic kinetic transport, which is related to the solute concentration under the oxidation temperature and time. Due to the higher content of Fe and Ni on the coating surface, these two elements are oxidized first during the oxidation process, forming the oxide film. This oxide film hinders further diffusion of oxygen into the coating interior to a certain extent. With the substantial consumption of Fe and Ni on the coating surface, an Fe- and Ni-depleted region forms, providing favorable conditions for the diffusion of other metallic elements to this region. As the oxidation process deepens, elements such as Nb, Al, V, and Ti are gradually oxidized, forming corresponding metal cations. These metal cations can act as high-valence dopants entering the oxide film, increasing the vacancy concentration in the oxide film and thereby accelerating the mutual diffusion of oxygen ions and metal ions.

### 3.3. Thermal Shock Performance

**Figure 6:**
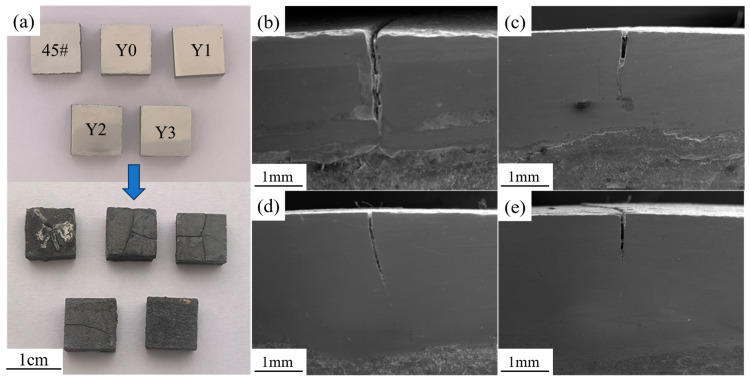
Macroscopic surface morphologies of samples before and after thermal shock, and microscopic morphologies of side surface cracks after thermal shock: (**a**) macroscopic morphologies; (**b**) Y0; (**c**) Y1; (**d**) Y2; (**e**) Y3.

Figure 6a shows the surface crack morphologies of the four composite coatings and the 45# steel substrate after the thermal shock experiment. It can be clearly observed that the 45# steel substrate exhibits extensive cracking on the surface, with poor surface quality. The Y0 and Y1 coatings show obvious network cracks on the surface. The Y2 coating has fewer cracks on the surface, but they almost penetrate the entire coating surface. In contrast, the Y3 coating only exhibits fine microcracks at the edges. Figure 6b–e display the microscopic morphologies of cracks on the side surfaces of the coatings, revealing cracks of varying degrees in the coatings. The Y0 coating exhibits wide and deep longitudinal cracks that nearly penetrate the entire cladding layer; with increasing Y_2_O_3_ content, the crack lengths gradually shorten, and the widths narrow.

**Figure 7:**
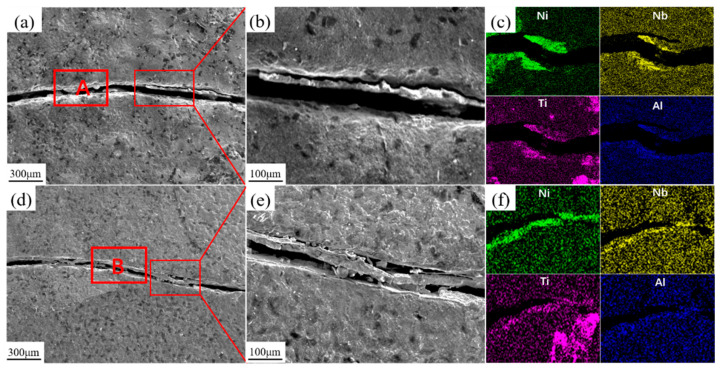
Microscopic morphologies of surface cracks on Y0 and Y3 samples after thermal shock and EDS distribution maps: (**a**–**c**) Y0; (**d**–**f**) Y3.

EDS scans were performed on the surface cracks of the Y0 and Y3 coatings, as shown in Figure 7. Both sides of the cracks contain high levels of Nb, Ni, Ti, and Al elements. This elemental distribution pattern is similar to that of the lamellar inter-dendritic structures, indicating that the coating cracks preferentially propagate and extend in the enriched regions of lamellar precipitated phases between dendrites. Throughout the entire thermal shock experiment, each high-temperature holding stage triggers high-temperature oxidation reactions in the coating material. The multi-component elements in the coating react with oxygen at high temperatures to form corresponding oxides. As the alloy composition on the coating surface is continuously consumed, pores and loose porous regions gradually form on the surface. Furthermore, due to differences in thermal expansion coefficients among different types and quantities of oxides, the propagation speed and size of cracks in the composite coatings exhibit significant differences.

### 3.4. Mechanical Properties

**Figure 8:**
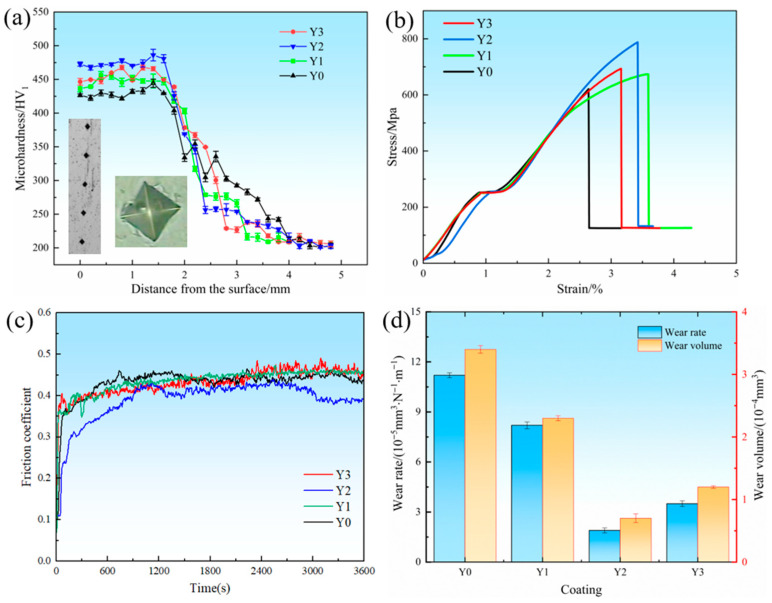
Microhardness and friction-wear data: (**a**) microhardness curves; (**b**) stress–strain curve; (**c**) friction coefficient curves; (**d**) mass loss and wear volume.

Figure 8a shows the microhardness curves of the cross-sections of the four coatings and the typical micro-indentation morphologies. The indentation boundaries are clear, presenting a typical parallelogram structure, with the diagonal lengths all maintained at approximately 20 μm. It is observed that with the increase of Y_2_O_3_ content, the microhardness first increases and then decreases. The average hardness of the Y2 coating reaches 467 HV. Next are the Y3 coating at 453 HV and the Y1 coating at 441 HV, both significantly higher than that of the Y0 coating at 428 HV. The addition of Y_2_O_3_ refines the coating structure and improves the coating’s density, thereby enhancing the coating’s hardness. However, an excessive amount of Y_2_O_3_ reduces the cooling rate of the melt pool and increases the dilution rate of the substrate [35,36], weakening the fine-grain strengthening effect and resulting in a decrease in microhardness.

Figure 8b shows the stress–strain curves of the coatings with different Y_2_O_3_ additions. Among them, the Y2 coating exhibits the highest tensile strength, followed by the Y3 and Y1 coatings, while the Y0 coating shows the lowest strength. As the Y_2_O_3_ content increases from 0 wt.% to 0.4 wt.%, the tensile strength of the coatings increases from 616 MPa to 673 MPa and 788 MPa, indicating that an appropriate amount of Y_2_O_3_ can significantly enhance the mechanical performance of the coatings. However, when the Y_2_O_3_ content is further increased to 0.6 wt.%, the tensile strength decreases to 693 MPa. According to the previous analysis, the addition of Y_2_O_3_ can refine the grain structure of the coating and increase the proportion of grain boundaries, which act as effective barriers to dislocation motion. This leads to an intensified work-hardening effect during plastic deformation, thereby improving tensile strength. Therefore, the enhancement in strength can be attributed to the combined effects of grain refinement and enhanced strain-hardening behavior induced by Y_2_O_3_ addition. However, when the addition of Y_2_O_3_ increases to 0.6%, the fluidity of the liquid metal decreases, resulting in a reduction in the integrity of the crystal structure. The formation of lamellar structures between dendrites provides preferential sites for crack initiation and propagation, which ultimately deteriorates the overall tensile strength of the coating.

Figure 8c shows the friction coefficients of the various coatings. It can be observed that the composite coatings exhibit a two-stage evolution in the friction coefficient during the wear process, characterized by an initial rapid increase followed by a gradual stabilization. This behavior occurs because, in the early stage of friction, the contact is primarily dominated by mechanical interlocking and plowing effects, which induce severe plastic deformation and local material delamination. The accumulation and detachment of wear debris lead to fluctuations and a sharp rise in the friction coefficient. At this stage, a stable oxide film has not yet formed, resulting in a high proportion of direct contact between the friction pairs and consequently a relatively large and rapidly increasing friction coefficient. As the friction process continues, the system gradually enters a stable friction stage. The continuous frictional heat promotes the formation of a dense oxide film on the coating surface, which serves as a protective layer and facilitates smoother sliding between the counterparts, thereby stabilizing the friction coefficient. In general, a higher friction coefficient tends to correspond to a greater wear loss [37]. The average friction coefficients of the Y0, Y1, and Y2 coatings show a gradually decreasing trend, with values of 0.451, 0.436, and 0.4, respectively. However, as the Y_2_O_3_ content increases to 0.6 wt.%, the average friction coefficient of the Y3 coating increases again to 0.433. Combined with the microhardness results, due to the reduced hardness of the Y3 coating, according to Archard’s law, its wear resistance also decreases [38,39]. Therefore, it exhibits the increased wear rate and wear volume as shown in Figure 8d.

**Figure 9:**
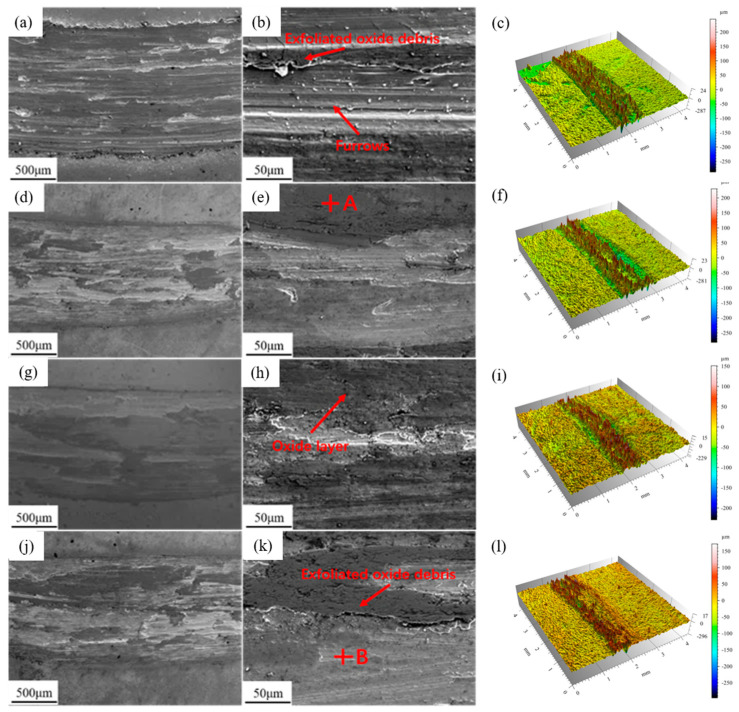
Microscopic morphologies after high-temperature friction and wear: (**a**–**c**) Y0; (**d**–**f**) Y1; (**g**–**i**) Y2; (**j**–**l**) Y3.

The SEM micromorphology and three-dimensional morphology of the four types of coating worn surfaces are shown in Figure 9. As can be seen from Figure 9a, the wear marks of the Y0 coating are relatively wide, and a large number of plowing grooves of varying depths appear on the worn surface. In Figure 9b, scattered debris is shown, and the phenomenon of oxide film peeling is quite obvious. This is mainly due to the high hardness of the friction pair, which, under the combined action of compressive stress and shear stress, exerts a plowing effect on the coating surface. These grinding swarf are formed by repeated squeezing and friction of the oxidized products that have fallen off. The oxide film is damaged under the action of shear force and continues to form abrasive wear on the grinding pair. It can be known from the three-dimensional morphology in Figure 9c that friction causes adhesive wear in the coating. Therefore, the wear mechanism of the Y0 coating is mainly the combined effect of abrasive wear and adhesive wear. In Figure 9d,g,j, the wear marks of the coating with added Y_2_O_3_ are narrower, and the oxide debris is significantly reduced. Compared with the Y0 coating, no deeper furrow morphology was found inside the wear scar, but slight adhesive wear was still observed, as shown in Figure 9f,i,l.

Table 2 shows the components of points A and B. The oxygen content in the dark area at point A is relatively high, and a partial oxide film has already formed on the surface. These oxide layers act as barriers, isolating the surfaces of the friction pairs, reducing direct metal contact and oxidative wear. The oxide film on the surface of Y3 is significantly less than that of the Y2 coating. The oxygen content in the light-colored area at point B is relatively low, indicating that the oxide film has not yet formed. Its wear resistance is poorer compared to Y2. This is mainly attributed to two aspects. One is the excessive addition of Y_2_O_3_ in the Y3 coating, which leads to an increase in the dilution rate between the coating and the substrate, resulting in a decrease in hardness. On the other hand, excessive Y_2_O_3_ leads to the agglomeration of Nb and Ti phases at the grain boundaries, increasing the brittleness of the grain boundaries. During the wear process, it is easy for brittle fractures to expand along the direction of surface microcracks, and the wear resistance decreases. The grain refinement of the Y2 coating enables the rapid formation of a dense oxide film during high-temperature friction and wear, which plays a lubricating role, resulting in less wear debris and reducing the further expansion of wear marks and the formation of deeper furrows.

## 4. Conclusions

This study prepared Ni_50_(AlNbTiV)_50_-xY_2_O_3_ composite coatings using laser cladding technology. Through characterization of the microstructures and high-temperature performance with different Y_2_O_3_ addition amounts, the following conclusions were drawn:

(1) Adding an appropriate proportion of Y_2_O_3_ to the complex concentrated alloy can refine the grains, but excessive addition leads to a slowdown in the molten pool cooling rate, resulting in larger grains and increased precipitation of lamellar structures in the interdendritic regions.
(2) In the high-temperature oxidation and thermal shock tests, the addition of 0.6 wt.% Y3 coating exhibits the optimal high-temperature stability. This is attributed to the higher density of lamellar precipitate phases rich in Nb and Ti between the dendrites in Y3, which possess lower oxygen diffusion coefficients and free energy.
(3) In the mechanical property test, the addition of 0.4 wt.% of the Y2 coating significantly improved hardness, tensile properties, and wear resistance through the fine-grain strengthening mechanism. The addition of excessive Y_2_O_3_ will increase the dilution rate of the substrate, and the lamellar precipitated phase will increase, resulting in a decrease in microhardness and tensile properties. Due to the decrease in hardness, during the cyclic load-friction process of applying pressure, the oxide film is more likely to fall off, which will instead reduce the strength of the coating.

## Figures and Tables

**Table 1:** Point scan results of elemental compositions at different positions in the composite coatings (wt.%).

Elements	Ni	Al	Nb	V	Ti	Fe
A	24.89 ± 1.24	2.31 ± 0.12	2.94 ± 0.15	5.71 ± 0.29	2.30 ± 0.12	61.8 ± 3.09
B	18.9 ± 0.95	1.50 ± 0.08	22.7 ± 1.14	3.95 ± 0.20	6.41 ± 0.32	46.4 ± 2.32
C	20.8 ± 1.04	1.76 ± 0.09	20.0 ± 1.00	4.25 ± 0.21	5.20 ± 0.26	47.8 ± 2.39

**Table 2:** Elemental compositions at different positions in the coating after high-temperature friction and wear (wt.%).

Elements	Ni	Al	Nb	V	Ti	Fe	O
A	22.47 ± 1.12	3.18 ± 0.16	10.63 ± 0.53	5.52 ± 0.28	6.13 ± 0.31	19.90 ± 1.00	32.17 ± 1.61
B	26.33 ± 1.32	1.42 ± 0.07	12.74 ± 0.64	7.34 ± 0.37	8.30 ± 0.42	24.81 ± 1.24	19.06 ± 0.95

## Data Availability

The original contributions presented in this study are included in the article. Further inquiries can be directed to the corresponding author.
